# Closed-Loop Crop Cascade to Optimize Nutrient Flows and Grow Low-Impact Vegetables in Cities

**DOI:** 10.3389/fpls.2020.596550

**Published:** 2020-11-12

**Authors:** Martí Rufí-Salís, Felipe Parada, Verónica Arcas-Pilz, Anna Petit-Boix, Gara Villalba, Xavier Gabarrell

**Affiliations:** ^1^Sostenipra Research Group (2017 SGR 1683), María de Maeztu Unit, CEX2019-000940-M, Institut de Ciència i Tecnologia Ambientals (ICTA-UAB), Universitat Autònoma de Barcelona, Barcelona, Spain; ^2^Department of Chemical, Biological, and Environmental Engineering, Universitat Autònoma de Barcelona, Barcelona, Spain; ^3^Chair of Societal Transition and Circular Economy, University of Freiburg, Freiburg, Germany

**Keywords:** cascade systems, nutrient recycling, urban agriculture, industrial ecology, urban metabolism

## Abstract

Urban agriculture systems can significantly contribute towards mitigating the impacts of inefficient and complex food supply chains and increase urban food sovereignty. Moreover, improving these urban agriculture systems in terms of nutrient management can lead to a better environmental performance. Based on a rooftop greenhouse in the Barcelona region, we propose a cascade system where the leachates of a tomato cycle from January to July (donor crop) are used as the main irrigation source for five successive lettuce cycles (receiving crop). By determining the agronomic performance and the nutrient metabolism of the system, we aimed to define the potential of these systems to avoid nutrient depletion and mitigate eutrophication, while scaling the system in terms of nutrient supply between the donor and the receiving crops. The results showed that low yields (below 130 g per lettuce plant) are obtained if a cascade system is used during the early stage of the donor crop, as the amount of nutrients in donor’s leachates, specially N (62.4 mg irrigated per plant in the first cycle), was not enough to feed the lettuce receiving crop. This effect was also observed in the nutrient content of the lettuce, which increased with every test until equaling the control (4.4% of N content) as the leachates got richer, although too high electrical conductivity values (near 3 dS/m) were reached at the end of the donor crop cycle. Findings on the uptake of the residual nutrient flows showed how the cascade system was able to take advantage of the nutrients to produce local lettuce while mitigating the effect of N and P in the freshwater and marine environments. Considering our case study, we finally quantified the scale between the donor and receiving crops and proposed three major ideas to optimize the nutrient flows while maintaining the yield and quality of the vegetables produced in the receiving crop.

## Introduction

Cities cover 3% of the Earth’s surface area, but host 55% of the world’s population (United Nations, 2014; SEDAC, 2016). The global nutritional demand thus concentrates in urban areas, resulting in long and complex supply chains. Urban agriculture (UA) has arisen as a promising solution that brings production closer to consumption points. However, UA is not free of environmental impacts. Similar to conventional agricultural systems, nutrient discharge resulting from intensive fertilizer use is particularly problematic due its impact on water eutrophication (Muñoz et al., 2008; Torrellas et al., 2012; Romero-Gámez et al., 2014, 2012; Boneta et al., 2019). The application of circular economy strategies in UA systems could help mitigate these impacts. Closing the nutrient cycles in soilless systems maintains the utility and value of scarce resources (Bocken et al., 2017), produces a regenerative effect on the environment (Ellen MacArthur Foundation and McKinsey Center for Business and Environment, 2015), and contributes to a reduction in water and fertilizer consumption (Carmassi et al., 2005; Rufí-Salís et al., 2020).

Two main strategies can be found in the literature to close the nutrient cycles in soilless systems. First, recirculation systems use drained water and nutrients in the same crop. This setup helps to reduce water and nutrient losses by closing resource flows (Ahmed et al., 2000; Agung Putra and Yuliando, 2015). However, it demands additional infrastructure, which requires high investment costs (De Pascale and Maggio, 2005; García-Caparrós et al., 2018) and increases the environmental impacts of the system (Rufí-Salís et al., 2020). Moreover, the high salinity of the drained water can cause yield reduction if it is directly recirculated to the crop without treatment or partial discharge (Magán et al., 2008; Grewal et al., 2011).

Second, cascade systems use drained water and nutrients to irrigate another crop (Incrocci et al., 2003). Since nutrients tend to reach high concentrations in the leachates and thus increase the salinity of this water flow, the salt tolerance of the receiving crop has been the main field of inquiry in the literature. For instance, Shannon et al. (2000) analyzed the salt tolerance of nine leafy vegetables using salty water to simulate drainage water, finding that swiss chard was the most salt tolerant among those evaluated. With a similar method, Grieve and Suarez (1997) focused on potential sulfate and selenium toxicity, concluding that purslane (*Portulaca oleracea*) is an excellent candidate for saline drainage water reuse systems. Real case studies with cascade systems were analyzed by Muñoz et al. (2017), who assessed the performance of beef tomato in a greenhouse as the donor crop and a sequence of lettuce, tomato, and endive as receiving crops. This study underlined the need to further study the observed yield decrease and its causes. Incrocci et al. (2003) assessed a cascade cropping system using tomato as the donor crop and cherry tomato as the receiving crop, highlighting the potential of cascade set-ups to increase the water use efficiency of the system. Nonetheless, little is known about the potential of a cascade system to diminish the nutrient load of drainage flows. García-Caparrós et al. (2016) explored this potential with a pot experiment using ornamental *Juncus acutus*. The authors found that the species used presented a good bioremediation potential based on the uptake of nitrogen (N) and phosphorus (P). García-Caparrós et al. (2018) also explored this potential using horticultural melon as the donor crop and ornamental rosemary as the receiving crop, finding a decrease in yield in the receiving crop, but also a great potential to optimize the water flows and remove the nitrates of the drained water. To our knowledge, there are no references in the literature exploring the potential of horticultural species (both as donor and receiving crops) to produce vegetables while mitigating nutrient depletion through the use of a cascade system and the quantification of the nutrient flows.

The present article aims to tap the full potential of cascade systems to produce local-grown vegetables in the framework of UA while diminishing the nutrient load by closing nutrient cycles in the framework of urban metabolism. Based on the agronomic and nutritional performance of five successive receiving crops of lettuce (*Lactuca sativa, Maravilla*) irrigated by a long-cycle donor crop of tomato (*Lycopersicon esculentum, Arawak*), we determined the feasibility and nutritional implications of cascade systems and provide recommendations to further improve the performance of this setup. This will enable a better implementation of UA systems in cities and inform decision-makers about the main benefits and improvement potential of reusing nutrients in UA by considering the principles of a circular economy.

## Materials and Methods

### System Under Study, Crop Description, and Experimental Design

The study was performed in a rooftop greenhouse (RTG) located on the ICTA-ICP building (41.497681N, 2.108834E) on the campus of the Universitat Autònoma de Barcelona, 15 km west of Barcelona, in the West Mediterranean region of the Iberian Peninsula. The crops grown in the greenhouse used rainwater harvested from a roof surface of 400 m^2^ (plus 500 m^2^ from the neighboring building). The irrigation system was hydroponic, where the substrate bags are filled with perlite with a pH of 7, an electrical conductivity of 0.09 dS⋅m^–1^, and a granulometry of [0–6].

The RTG section used for the donor tomato crop was southeasterly facing and occupied an area of 84.3 m^2^. 57 substrate bags were used (40L), planting 3 seedlings per bag, totaling 171 plants. The tomato crop was planted on January 14th and uprooted on August 2nd, 2019. The average concentration of fertilizers in kg/m^3^ was KPO_4_H_2_ – 0.283, KNO_3_ – 0.138, K_2_SO_4_ – 0.367, Ca(NO_3_)_2_ – 0.533, CaCl_2_ H_2_O – 0.133, Mg(NO_3_)_2_ – 0.178, Hortilon – 0.011, and Sequestrene – 0.011, although the nutrient solution was adapted based on the evolution and phenological stages of the tomato crop. The leachates of the tomato crop were collected through connected pipes from leachate trays and transported to a 300-L tank using a submergible pump.

**FIGURE 1:**
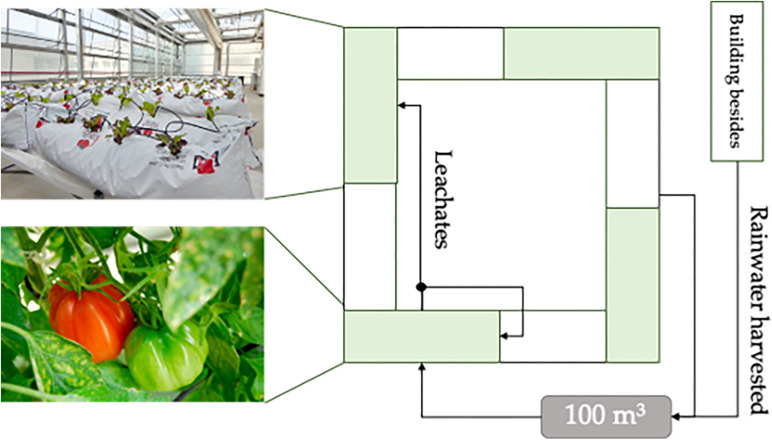
Diagram of the cascade system.

To maximize the nutrient recycling, these leachates were used to irrigate two crops. First, the same tomato crop using a recirculation system (totaling 7.5 m^3^), whose performance was left out of the study to focus only on the cascade system. Second, a receiving lettuce crop located in another section of the RTG southwesterly facing using 2 L/h drippers (totaling 8.8 m^3^). Figure 1 shows the diagram of the water flows of the system.

Five successive lettuce cycles were grown as shown in Table 1. The choice of lettuce was based on the lower nutritional demand from this crop compared to tomato. Low nutrient concentrations were expected in the leachates of the tomato crop, and thus lettuce can potentially produce competitive yields with the nutrients supplied through the cascade system. Average temperatures and relative humidity per test are shown in Supplementary Figures S1, S2, respectively.

**TABLE 1:** Number of plants and calendar of tests undergone in the present study.

**Test**	**Treatment**	**Planted**	**Harvested**	**Plant number**
T1	Cascade	February 8	March 5	128
T2	Cascade	March 5	April 11	128
T3	Cascade	April 24	May 31	128
	Control			64
T4	Cascade	May 31	July 1	128
	Control			64
T5	Cascade	July 1	August 1	128
	Control			64

Plants were distributed in 4 substrate trays, with 8 perlite bags each using a 4 × 2 organization and with a 1.2-m separation between trays. Each substrate bag can allocate 4 plants, totaling 32 plants per tray. All campaigns were planted from nursery plants.

Besides campaigns three to five of the cascade system, we planted mirror control crops using the same species and variety to compare the results in terms of yield and nutrient content during the seasonal period with expected higher water demand and salinity. We used 2 substrate trays for the control, totaling 64 plants.

### Water and Nutrient Monitoring

To assess the water flows in the cascade system, analogic flow meters were coupled to the water system. Samples of the irrigation in the receiving crop and the control were collected directly from the drippers placed in the perlite bags. To determine the concentration in the water flows, the respective samples were collected three times per week and analyzed using ion chromatography (ICS-1000 and AS-DV by Dionex) to obtain concentration results for the anions nitrite (NO_2_^–^) and nitrate (NO_3_^2–^). The same samples were externally analyzed using ICP-OES atomic spectroscopy (Optima 4300DV by Perkin-Elmer) to obtain results for P, potassium (K), magnesium (Mg), calcium (Ca), and sulfur (S). Nutrient content in the lettuce was determined based on three plants randomly selected at the end of the harvest. Samples were sorted into envelopes and dried in a furnace at 65°C for 1 week before analyzing externally the concentration of P, K, Ca, Mg, and S via ICP-OES atomic spectroscopy (Optima 4300 DV by Perkin Elmer) and the concentration of N with elemental analysis (Flash EA 2000 CHNS by Thermo Fisher Scientific). Since the sample for every test and treatment is 3 random plants analyzed twice, a formal statistical analysis was not performed due to the low sample size.

To calculate the accumulated irrigated nutrients per test, the concentration values obtained through the continuous analysis of the water samples for each nutrient were multiplied by the amount of water irrigated between the date a specific sample was taken and the date of the previous sample. The values obtained were then aggregated to obtain the final accumulated irrigated nutrients per test, and divided by the number of plants in a specific test to obtain this parameter per plant.

### Statistical Methods

A set of statistical methods and tests were used to verify and strengthen the results obtained in different sections. The tests were performed through the use of different packages developed for R programming software, considering a statistical significance when *p*-values were less than 0.05.

Shapiro Wilk’s test (Shapiro and Wilk, 1965) was used as the method to analyze the normality of observations or residuals obtained through linear regressions. Levene’s test (Levene, 1961) was used as the method to analyze the homogeneity of variances. Levene’s test is an alternative to Bartlett’s test with less sensitivity to non-normal data. If the two previous tests (Shapiro-Wilk’s and Levene’s) presented *p* > 0.05, we applied an analysis of covariance (ANCOVA) to the linear regression to evaluate if the means of a dependent variable were equal under different levels of an independent variable. Oppositely, we applied Kruskal-Wallis test (Kruskal and Wallis, 1952) as the non-parametric analog of ANCOVA, recommended with non-normal distributions and inequality of variances (Feir-Walsh and Toothaker, 1974) to check if samples came from the same distribution.

Three different methods were used to compare the statistical significance between slopes of a linear regression. The first one was based on Meseguer-Lloret (2017b) for homogeneous variances and Meseguer-Lloret (2017a) for non-homogeneous variances, and is based on a *t*-test. This method requires a prior test for normality. If a normal distribution is observed, a F-test can be performed to determine if the variances between treatments were homogeneous, since it is accurate only for normal data. However previous research concluded that the *t*-test is highly robust for equal sample size (Welch, 1938; Milton and Tsokos, 1983), which is the case for most data in this study. Based on the method described by Meseguer-Lloret (2017a) for non-homogeneous variances, a *t*-test should be performed by calculating t_*calc*_ as expressed in Eq. 1 and t’_*calc*_ as expressed in Eqs 2–4, being “b” the value of the slope, “s^2^” the variance, and “t” the specific value in the *t*-table of probabilities.

(1) $$t_{c\,a\,l\,c}=\frac{\left|b_{1}-b_{2}\right|}{\sqrt{s_{b_{1}}^{2}+s_{b_{2}}^{2}}}$$

(2) $$t_{c\,a\,l\,c}^{\prime }=\frac{t_{1}\cdot s_{b_{1}}^{2}+t_{2}\cdot s_{b_{2}}^{2}}{s_{b_{1}}^{2}+s_{b_{2}}^{2}}$$

(3) $$t_{1}=t_{95\%}^{n_{1}-2}$$

(4) $$t_{2}=t_{95\%}^{n_{2}-2}$$

If t_*calc*_ > t’_*calc*_, the slopes analyzed are statistically different. On the other hand, based on the method described by Meseguer-Lloret (2017b) for homogeneous variances, a *t*-test should be performed to calculate t_*calc*_ as expressed in Eq. 1 and t_*tab*_, which is the value in the *t*-table with a 95% of probability and “n_1_ + n_2_ -2” degrees of freedom, as expressed in Eq. 5.

(5) tt⁢a⁢b⁢ 95%=t95%n2-2

If t_*calc*_ > t_*tab*_, the slopes analyzed are statistically different. Oppositely, the slopes analyzed are statistically similar if t_*calc*_ < t_*tab*_.

The second method is based on Andrade and Estévez-Pérez (2014). Although the authors state that the *t*-test is a robust method to compare the statistical difference between slopes, they suggest the use of the ANCOVA as a simpler method.

The third method consists on the analysis of the “Estimated Marginal Means Of Linear Trends” through the “emtrends” function from the “emmeans” R package (Lenth et al., 2020). As stated by Lenth et al. (2020), “*emtrends is useful when a fitted model involves a numerical predictor interacting with another predictor.*”

## Results and Discussion

This section presents and discusses the results divided in different sections: see section “Production,” section “Water: Irrigation,” section “Water: EC and pH,” section “Water: Nutrient Content,” section “Biomass: Nutrient Content,” section “Decreasing Eutrophication Potential and Nutrient Depletion, and section “Scaling of the System.” Values in X ± Y form express average ± standard deviation.

### Production

**FIGURE 2:**
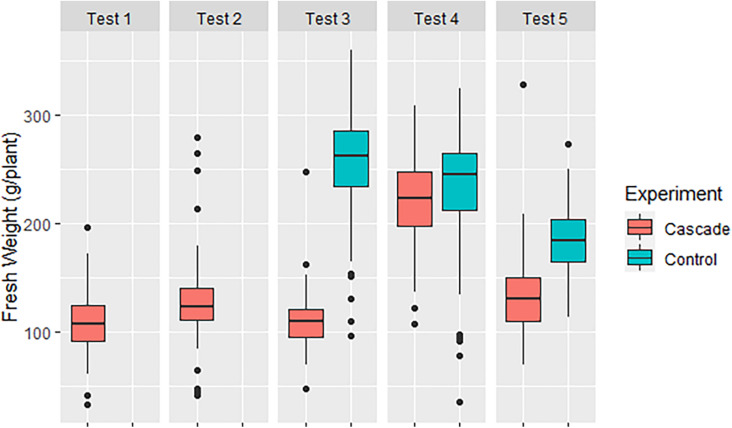
Production of the different tests.

Figure 2 shows the average fresh weight per lettuce in every test. We can see that Tests 1 (February) and 3 (May), produced low and similar yields: 108.66 ± 26.85 and 109.82 ± 22.39 g/plant, respectively. Test 2 (April) produced slightly more yield with a higher variability of data (127.95 ± 34.60 g/plant), while Test 4 (June) and Test 5 (July) exerted the highest yields, with 220.31 ± 38.27 and 134.17 ± 34.84 g/plant, respectively. On the other hand, the control treatments in Test 3, 4, and 5 (254.91 ± 52.66, 232.49 ± 62.25, and 185.11 ± 32.75 g/plant) always had more weight than its respective cascade crops. The low yields obtained in the receiving crop in the cascade system coincide with the findings by Muñoz et al. (2012), who used tomato as both donor and receiving crop and Muñoz et al. (2017), who used tomato as donor crop and lettuce as receiving crop.

We determined that only cascade’s Test 1 (*p* = 0.78) and 4 (*p* = 0.26) and control’s Test 5 (*p* = 0.83) presented a normal distribution (*p* > 0.05). However, a normal distribution was observed when the test was done including all the control data (*p* = 0.22), but not when analyzing the entire cascade dataset (*p* < 0.05). Both of these results were expected based on the homogenous irrigation in the control and the foreseeable variability in nutrient content of the leachates of the donor crop, analyzed in-depth in section “Water: Nutrient Content”. When normality is studied for the residuals obtained through multiple combinations between tests and treatments, a non-normal distribution is observed in all of them (*p* < 0.05). On the other hand, the biggest differences between treatments were found in Test 3 and Test 5, in which the control produced 132 and 37% more yield, respectively, than their simultaneous cascade tests. Oppositely, the differences between treatments in Test 4 were narrowed to 6%. Despite this big variability, none of the comparisons between treatments (i.e., Control vs. Cascade in Test 3, 4, and 5) presented a significant homogeneity of variances (*p* < 0.05). Based on the non-normality detected, we applied Kruskal-Wallis test to compare between treatments. As expected, the results showed that the distribution differences between cascade and control were significant in every test (*p* < 0.05).

### Water: Irrigation

**FIGURE 3:**
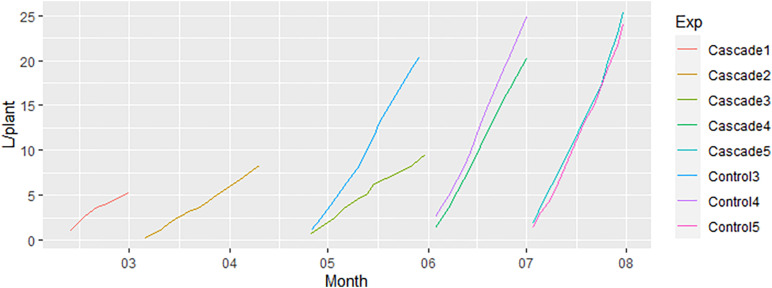
Irrigation per plant of the different tests.

All water irrigated to the receiving crop (8.8 m^3^) was supplied through the leachates of the donor crop, thus being a residual flow that the lettuce crop is taking benefit from and avoiding the use of new water. Irrigation was increased in every successive crop based on the climatic conditions inside the greenhouse, irrigating 5 times more water in Test 5 than in Test 1 (Figure 3). The slope of the linear regression in Test 1 and 2 of the cascade treatment was similar (0.22 and 0.23 with *R*^2^ of 0.96 and 1.00, respectively). This minimum difference was expected considering the temperatures reached inside the greenhouse during Test 1 and 2 periods, which were really similar (Supplementary Figure S1). We observed an anomaly in the water irrigated between the cascade system and the control in Test 3. Control Test 3 irrigated 20.30 L/plant with a slope of 0.60 (*R*^2^ = 1.00), doubling the slope of cascade Test 3 (0.24 with *R*^2^ = 0.99), that irrigated 9.60 L/plant. The statistical differences between the slopes obtained in the different tests and treatments were determined through a multi-step method with a final *t*-test. First, we determined if the individual accumulated irrigation per plant, test and treatment followed a normal distribution, which was confirmed for all of them (*p* > 0.05). A normal distribution was also confirmed for the residuals (*p* > 0.05). In the next step, the variances between treatments were labeled as non-homogeneous in Test 3 (*p* < 0.05) and homogeneous in Test 4 and 5 (*p* > 0.05). Considering the non-homogenous variances in Test 3 we calculated t_*calc*_ and t’_*calc*_, obtaining the values 25.85 and 2.22, respectively. Considering that t_*calc*_ > t’_*calc*_, we concluded that the slopes between cascade and control in Test 3 were statistically different. On the other hand, we performed a *t*-test for homogeneous variances for Test 4 and 5. t_*tab*_ values for Test 4 and 5 were both 1.71. t_*calc*_ for Test 4 was 6.38, while a value of 0.14 was obtained for Test 5. Given that t_*calc*_ > t_*tab*_ for Test 4, the slopes between treatments in this test were observed to be statistically different. Oppositely, the slopes between treatments in Test 5 were statistically similar since t_*calc*_ < t_*tab*_.

The ANCOVA applied to the data in Figure 3 strengthened the statistical results obtained in the *t*-test since the same conclusions were obtained: differences between slopes in Test 3 and 4 were found to be statistically different, while in Test 5 were observed to be statistically similar.

To double-verify the statistical conclusions, we analyzed the “Estimated Marginal Means Of Linear Trends.” The outcome of the analysis was the same as the one obtained through the *t*-test and ANCOVA: statistically differences between slopes in Test 3 and 4 (*p* < 0.05) and statistically similar slopes for Test 5 (*p* = 0.89) between cascade and control treatments.

The difference in irrigation rate in Test 3 was related to a shortening in the amount of leachates generated by the tomato crop, coupled with the parallel irrigation with leachates in the donor crop. To solve this problem, three options were considered.

First, to increase the irrigation in the donor crop, thus leaching more water that would be available for the receiving crop. This option was discarded because increasing the irrigation water use efficiency (assumed to be the volume of irrigated water applied per unit of yield) of the receiving crop while decreasing this parameter for the donor crop seemed contradictory. Moreover, the composition of the leachates will likely vary if more water is used in the donor crop. Second, to add water to the cascade system to meet the demand of the receiving crop. This option was also discarded because it would imply a modification in the composition of the irrigation of the receiving crop, thus blurring the effect of a cascade system. More important, both of these strategies were contradictory with the general aim of cascade systems: to take advantage of the residual flows of water and nutrients. If changes in the predefined system are needed, the value of the implementation of cascade systems decrease drastically. What could be seen as the best strategy is based on the practitioner or farmer priorities. When there is shortage of water, the farmer should prioritize in which system the residual flows are used. With a cascade set-up, the receiving crop can be prioritized during water shortage periods, minimizing the recirculation of water in the donor crop itself. However, if the donor crop is already not recirculating its own water and there is still water shortage, the first and second options outlined in this section can be applied considering the mentioned limitations.

The differences between treatments in Test 4 and 5 were narrower than the ones observed in Test 3 since there were not episodes of water shortening. Control Test 4 presented a slope of 0.82 (*R*^2^ = 0.99), 18% higher than the slope for cascade Test 4 (0.69; *R*^2^ = 1.00). Slope for Test 5 was the same for both treatments (0.81; *R*^2^ = 0.99).

### Water: EC and pH

**FIGURE 4:**
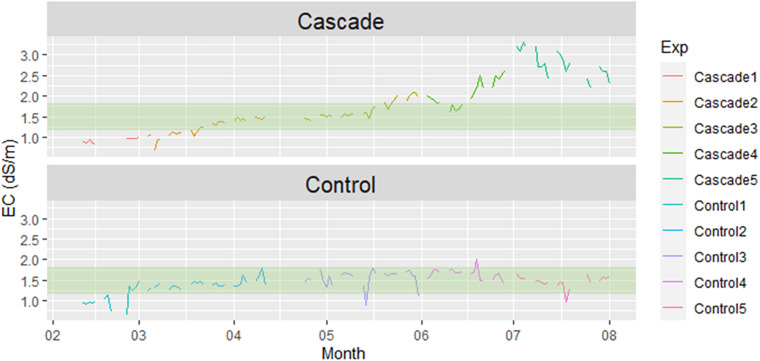
Electrical conductivity (EC). Highlighted area represents the suitable EC range to grow hydroponic lettuce stated by Singh and Dunn (2016).

Figure 4 shows the electrical conductivity (EC) values for the entire period under study. Since the control irrigation was manually controlled through the addition of fertilizers based on crop requirements, the EC values showed a great stability. On the other hand, the EC of the water used to irrigate the cascade crops increased over time, as the leachates of the tomato crop got richer in nutrients. The highest value was reached in the final cascade cycle, with 2.78 dS/m, much higher than the irrigation of the control (1.46 dS/m). Abou-Hadid et al. (1996) states that the perfect EC value for growing lettuce falls within the 1.0–1.5 dS/m range. Other literature specific for hydroponic lettuce mention that slightly higher values (1.2–1.8 dS/m) could be beneficial (Singh and Dunn, 2016). Most experiments found in the literature report greater yields within these values (Serio and Elia, 2001; Miceli et al., 2003; Samarakoon et al., 2019). Among the cascade test performed in our study, Test 1 (0.92 dS/m), Test 2 (1.25 dS/m) and Test 3 (1.68 dS/m) fell within the optimal range reported by Singh and Dunn (2016) (Figure 4 – highlighted area). EC readings for drainage water in cascade systems are higher than the ones observed in Figure 1, such as 3.62 dS/m reported by García-Caparrós et al. (2016) in drained water of *Ruscus aculeatus*. The authors also reported that a dilution 1:2 with fresh water was able to decrease the EC to values <3 dS/m. In this sense, to compensate the high EC reached in the final cascade cycle, we added fresh water in the leachates, thus triggering the unstable values observed in Figure 3 for Test 5. However, EC values obtained in the present study weren’t as unsuitable as expected considering the focus on salt tolerance from the literature around cascade systems. Oppositely to EC, pH values decreased overtime for the cascade cycle, as shown in Supplementary Figure S3. A pH optimal range to grow hydroponic lettuce of 6.0–7.0 is reported by Singh and Dunn (2016), and of 5.6–6.0 by Brechner and Both (2013). Considering a broad range, all cascade tests presented optimal pH mean values except Test 1, that had a mean pH value slightly higher than 7.

### Water: Nutrient Content

**FIGURE 5:**
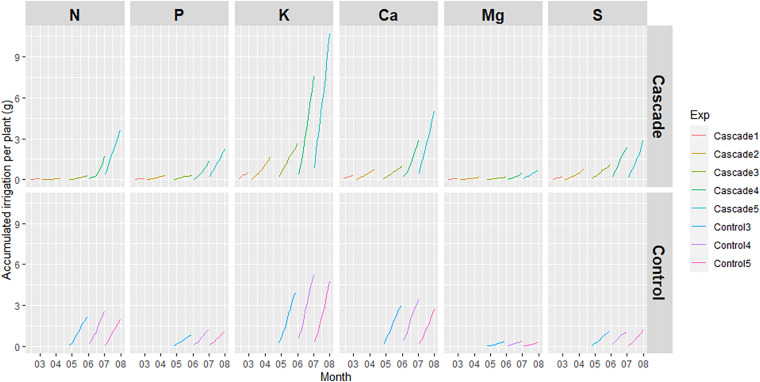
Accumulated irrigated nutrients per plant per test.

Figure 5 shows the accumulated irrigated nutrients per plant per test. As expected, the nutrients irrigated in the cascade treatment increased over time, coinciding with the amount of nutrients leached by the tomato crop. K was the nutrient with the highest quantities in the cascade irrigation (reaching 10.7 g/plant in Test 5 of the cascade system), followed by Ca and N, reaching 5.0 and 3.6 g/plant in Test 5, respectively. On the other hand, nutrients irrigated in the control treatment presented a similar behavior among tests.

Although the linear regressions observed in section “Water: Irrigation” regarding the irrigated water presented *R*^2^ values around 0.99, the evolution of the accumulated irrigated nutrients per plant (Figure 5) could also be affected by the concentration of nutrients in the water flows. As expected, the nutrient in the control irrigation followed a completely linear regression in all nutrients (*R*^2^ ≈ 0.99) since the nutrient solution supplied to the crop is completely homogenous. On the other hand, the linear regression of the accumulated irrigated nutrients in the cascade treatment highly fitted with the observations, with *R*^2^ values higher than 0.97. The only exception was N in Test 4, which presented a *R*^2^ = 0.85. The reason behind this behavior was the low quantities of this nutrient available through the leachates of the donor crop in Tests 1, 2, and 3. In this sense, we can see that this N limitation is overcome in the middle of Test 4 (concentration jumps from 35 to 111 mg/L), probably related to a N increase in the nutrient solution supplied to the donor crop. In this sense, the nutrient supply to the donor crop and its stability can be highlighted as a relevant parameter to also stabilize the nutrient content of the leachates and thus, provide a balanced and reliable source of nutrients to the receiving crop in a cascade system.

The slope of the accumulated irrigated nutrient per plant per test was compared between treatments for every specific nutrient through ANCOVA (Andrade and Estévez-Pérez, 2014). A prior step was done to assess the normality of the residuals, confirming the normality for all nutrients and tests apart from N in Test 3 with a *p* < 0.05. An analysis to determine the homogeneity of variances was applied to all combinations of nutrients and tests. As expected, Test 3 for N also presented statistically different variances (*p* < 0.05). Nonetheless, other cases were detected: P, K Ca and Mg for Test 3, S for Test 4 and K, Mg and S for Test 5. In this sense, ANCOVA was applied to the remaining cases with normal distribution and homogeneity of variances: Test 3 for S, Test 4 for N, P, K, Ca and Mg and Test 5 for N, P, and Ca, observing statistically different slopes between treatments in all of them (*p* < 0.05). The same outcome was obtained when analyzing the “Estimated Marginal Means Of Linear Trends” for these cases, although one exception was detected. P in Test 4 presented statistical different slopes through ANCOVA (*p* < 0.05), but statistical similarity was observed through “emtrends.”

### Biomass: Nutrient Content

**FIGURE 6:**
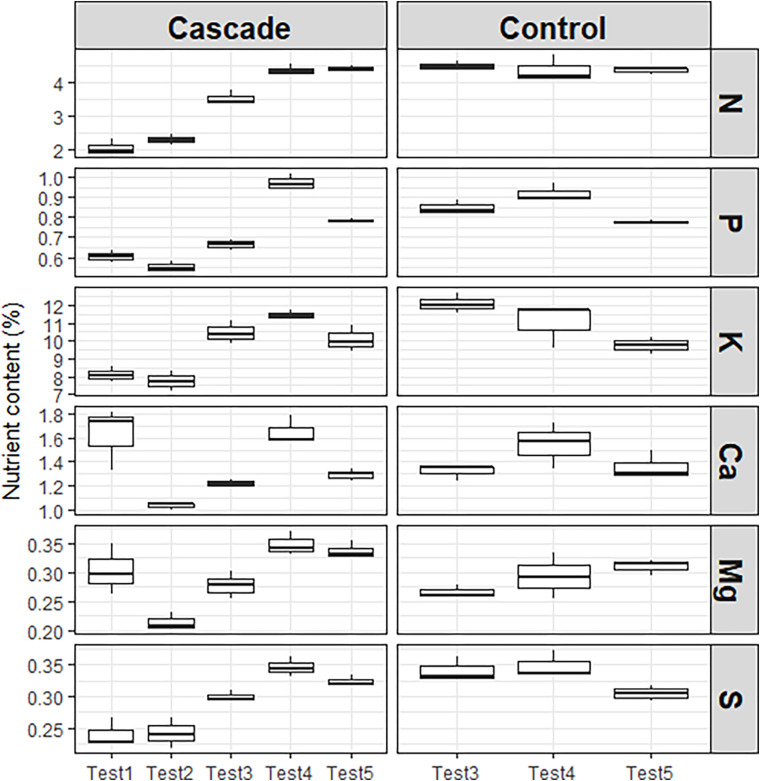
Nutrient context per plant (%).

Figure 6 shows the nutrient content in the lettuce for cascade and control tests. Every nutrient had a different behavior among tests, especially in the cascade treatment. N increased over time, especially between the 2nd and the 3rd test, and the 3rd and the 4th test. The 5th test presented the highest concentration of this nutrient. This tendency was expected based on the findings of the previous section, where we observed the increasing concentration of N in the leachates of the donor crop. Although the tendency related to the concentration in the water flow was observed for most nutrients, the 5th test was not the one with the highest nutrient content in the biomass for other nutrients analyzed. The 4th test was the one with the highest concentrations for P, K, Mg, and S with 0.97, 11.43, 0.34, and 0.35%, respectively. For Ca, the concentration in the 1st test was higher than the one observed for the 4th test (1.65%), which doesn’t correlate with its supply through the cascade system (Figure 5), and neither with the concentration of Ca in the leachates flow from the donor crop, which increased overtime. This high absorption of Ca in the first test neither correlates with traditional compatibilities/incompatibilities between nutrients (Mulder, 1956), since absorption of Ca may be depressed by excessive amount of Mg, which was also highly absorbed in Test 1, or favored with excessive nitrate concentrations, which was limitedly supplied through the cascade system in the first tests.

As expected, the control presented less differences between tests, especially for N. Due to differences in irrigated water between the cascade and the control in Test 3, nutrient content in the control was higher than in the cascade treatment for this test for all nutrients. When the irrigated water differences between the control and the cascade tests was normalized again in Tests 4 and 5, differences between treatments decreased. The average N content for the control (4.4%) was really similar to the one presented in tests 4 and 5 of the cascade system (4.4%). We could also find really similar concentrations between treatments in Test 4 for K (≈11.02%) and S (≈0.35%), and in Test 5 for P (≈0.78%), K (≈9.9%) and Ca (≈1.3%).

### Decreasing Eutrophication Potential and Nutrient Depletion

Table 2 shows the amount of nutrients that were taken up by the lettuce plants (Supplementary Table S1) with respect to the irrigated through the cascade system (Supplementary Table S2). Supplementary Table S3 shows the amount of nutrients leached by the lettuce plants. The ratio between nutrients taken up and irrigated decreased over time given the fact that the amount of nutrients being irrigated through the cascade system increased as the leachates from the tomato crop got richer. We can see that all N irrigated through the cascade system was taken up by the lettuce crop in tests 1 and 2, with two major consequences. First, a possible nitrogen deficiency that made Test 1 the one with the lowest yield, making the cascade set-up inefficient in terms of production. Second and with a bioremediation perspective, the removal of all N from the residual flow used to irrigate the receiving crop triggered the complete mitigation of the marine eutrophication impacts caused by the leachates of the donor crop. This mitigation decreased over time, getting down to 43.1% of removal in Test 3, and being only 6.5% in Test 5. The removal of P was already below one third in Test 1, thus just mitigating part of the freshwater eutrophication impacts. The removal rate of P got below 10% in Test 2, and it reached its lowest level in Test 5 with only 1.9%. Given the non-renewable nature of P, the reuse of P is not only relevant to avoid freshwater eutrophication, but also in terms of mitigating its depletion. K was the nutrient irrigated with the most quantity through the cascade system (Figure 4). However, high removal rates were only reached in Test 1 (63.4%). Removal rate in Test 2 already got below 25% and kept decreasing over time.

**TABLE 2:** Uptake (%) of irrigated nutrient per plant in cascade treatments.

**Nutrients**	**T1 – Cas (%)**	**T2 – Cas (%)**	**T3 – Cas (%)**	**T3 – Con (%)**	**T4 – Cas (%)**	**T4 – Con (%)**	**T5 – Cas (%)**	**T5 – Con (%)**
N	143.8*	115.9*	43.1	17.1	23.3	16.4	6.5	16.7
P	27.8	9.0	8.7	10.1	6.4	6.9	1.9	5.0
K	63.4	24.4	17.4	31.8	13.4	19.8	5.1	16.0
Ca	23.7	6.9	5.4	4.6	5.1	4.2	1.4	4.0
Mg	15.2	6.4	6.3	8.1	6.3	7.3	2.4	8.1
S	4.5	1.6	1.1	3.2	1.3	3.0	0.6	2.0

Cas, cascade system; Con, control. *Values above 100% mean complete uptake. Due to the small amount of nutrients leached by the donor crop during T1 and T2 the experimental and analytical error is increased.

Since the irrigation in the control was more controlled both in terms of water and nutrients, the removal rates among tests were more stable. This was specially the case of N, Ca, and Mg, while P slightly decreased in control tests 4 and 5, probably due to a combination of changes in the nutrient solution and an increase in P consumption of the donor crop in the production stage.

### Scaling the System

Other studies that considered the use of a the leachates of a donor crop to irrigate horticultural products did not account for the variability of nutrients along the cycle of the donor crop. For example, Choi et al. (2011) first collected the waste nutrient solution from a hydroponic tomato crop and then applied it to a cabbage crop instead of connecting a donor and a receiving crop simultaneously in a cascade system. Therefore, the nutrient concentration supplied by the authors was stable, avoiding problems related to variability but still subjected to possible nutrient limitations. However, NO_3_^–^ concentration in Choi et al. (2011) study (1285 mg/L) was always above all the NO_3_^–^ values determined in our study. Considering the higher nutrient content of lettuce compared to cabbage (Weber, 2016), it is not surprising that Choi et al. (2011) obtained higher yields than the control. In a similar experiment, Zhang et al. (2006) also obtained higher yields using tomato leachates in muskmelon and cucumber. By using the same donor crop, Incrocci et al. (2003) irrigated a cherry tomato cycle. Since the focus of the author was on the effect of salinity, the waste nutrient solution was adjusted in terms of nutrient content to meet the same amount as the control: 161 mg/L of N. This concentration was lower than the one used by Choi et al. (2011). However, this value was not reached in the cascade system of the present study until Test 4, in which the yield differences between the control and the cascade plants was the narrowest (Figure 2). With a cascade set-up using tomato as both donor and receiving crops, Muñoz et al. (2012) converge with the present study since the authors observed yield reductions in the receiving crop compared to the control [as also found by Muñoz et al. (2017)], irrigated with N concentrations of 198 and 310 mg/L, respectively. The N concentration in the tomato leachates in Muñoz et al. (2012) (198 mg/L) is more than two times the average concentration leached by the tomato donor crop of the present study (72.5 mg/L). This probably indicates excessive N in the irrigation of the donor crop, which could be adjusted without yield reductions as suggested and quantified by the same authors.

However, none of these authors considered the variability of nutrient content as a relevant parameter in cascade systems. Taking into account the need for a suitable dimensioning between donor and receiving crops in cascade systems to define best practices (Muñoz et al., 2017), we scaled the system in terms of nutritional demand of the receiving crop.

To quantify the scaling between donor and receiving crops in the system under study, we considered the average uptake of the control for all nutrients [N (4.4%), P (0.84%), K (11.0%), Ca (1.4%), Mg (0.29%), and S (0.33%)] and the average yield of the control (224 ± 58 g/plant). Table 3 shows the quantity of lettuce that could be grown with the maximum quantity of nutrients that could be supplied through the cascade system, considering that all leachates of the donor crop are applied to the receiving crop through the cascade system.

**TABLE 3:** Amount of lettuce that could be produced in the cascade system under analysis considering the application of all tomato leachates.

	**Units of lettuce plants/tomato plant**					**Units of lettuce plants/171 tomato plants**				
Nutrients	T1	T2	T3	T4	T5	T1	T2	T3	T4	T5
N	0.1	0.6	1.7	6.3	9.1	17.1	101.8	286.4	1073.5	1554.2
P	0.9	8.5	7.1	29.6	30.0	145.6	1460.9	1216.5	5065.1	5131.1
K	0.4	3.4	4.5	12.8	11.0	63.9	585.3	769.7	2188.8	1874.6
Ca	1.6	12.2	13.6	36.8	40.3	267.6	2091.5	2329.4	6288.4	6888.7
Mg	2.1	13.3	12.8	29.5	28.7	366.6	2275.7	2181.9	5036.6	4912.8
S	5.2	52.9	65.3	139.1	99.4	897.6	9042.9	11161.7	23779.4	17005.9

As we can see in Table 3, the limiting nutrient was always N, allowing to grow a 1:10 and a 6:10 system for Test 1 and 2, respectively. P (9:10) and K (4:10) in Test 1 were the other nutrients with less than a 1:1 ratio between the tomato donor crop and the lettuce receiving crop. Considering N as the limiting nutrient, we can state that in our system with 171 tomato plants, we could grow 17 lettuce plants in Test 1, while being able to grow 1554 lettuce plants in Test 5, thus giving the practitioners three main options in terms of crop management, considering an ideal situation where we have an area that can allocate 1554 lettuce plants, which is the number of lettuces that we could grow in Test 5 considering the N content in the tomato leachates.

First, to grow 17 lettuce plants in all tests. This option will ensure the production of the same amount of plants in every test but will present huge inefficiencies in terms of space (if the rest of the area is not occupied by other crops) and nutrient flows, discharging a great quantity of nutrients that will increase over time as the leachates of the donor crop become nutrient-richer. This nutrient inefficiency could be mitigated by applying recirculation in the donor crop itself. Second, to grow 17 (Test 1), 101 (Test 2), 286 (Test 3), 1073 (Test 4), and 1554 (Test 5) lettuce plants connected to the cascade system, while growing 1537 (Test 1), 1453 (Test 2), 1268 (Test 3), and 481 (Test 4) lettuce plants irrigated using a conventional nutrient solution. With this option, yield would be maximized, while adapting the nutrient demand of the lettuce cycles to the nutrient supplied through the cascade system without requiring further nutrient management. Third and finally, start with 1554 lettuce plants connected to the cascade system. To avoid nutrient deficiencies as the ones observed in the present study, the nutrient content in the leachates tank used to irrigate the receiving crop must be adjusted. A specific amount of all nutrients should be added in Test 1, while N, P and K should be added in Test 2 and 3, and only N should be added in Test 4. This option would be the most efficient to produce the same amount of lettuce over time while minimizing the nutrient input. However, it would require a detailed control of nutrient flows to avoid nutrient deficiencies. It is important to understand that, despite the strategies mentioned above, the list of management practices is endless: mix and store the leachates during Test 1 to increase the nutrient supply to Test 2 (and omit Test 1 of lettuce), increase the water irrigated to the donor crop, etc. However, the value of all possible strategies rely on their performance in real crops. Therefore, we highlight the need to evaluate and quantify the potential yield that could be reached through the application of these strategies. In addition, the analysis of the nutritional and water flows in pilot or full-scale experiments should be included in the list of upcoming challenges to verify that the plant nutrition is optimal and that the cascade system is well scaled to mitigate eutrophication impacts derived from the depletion of nutrients in the donor crop.

## Conclusion

The present paper has presented an evaluation of a cascade system with a long-cycle tomato donor crop and five successive cycles of lettuce. The assessment of the agronomic performance and the nutrient flows have shed light on the potential of these systems to mitigate nutrient depletion in cities while producing food in the framework of urban agriculture.

The variation of nutrient content of the leachates produced by the donor crop is a key parameter to plan the amount of plants that can be planted of the receiving crop. The early stage of the donor crop could only produce 0.1 lettuces per tomato plant, with N as the limiting nutrient. On the other hand, the late stage of the donor crop was able to leach enough nutrients to feed 9 lettuces per tomato plant. However, attention must be paid on the electrical conductivity of the water flow to stay within non-harmful values. Nevertheless, the cascade system was shown to be efficient to mitigate the nutrient discharge of open systems, especially in terms of N and P to avoid eutrophication impacts in the early stage of the tomato crop. To this end, a good scaling between the two crops of the system is vital to tap the full potential of the cascade set-up, while having different options in terms of system management.

Given the findings of this study, we encourage future researchers to test different kind of horticultural crops. Considering the nutritional problems in the beginning of the cycle of the donor crop and the harmful salinity that can be reached at the end, further research should test possible combinations of donor and receiving crops that minimize these two problems. Reporting the limitations of these kind of systems is key to a transparent process of decision-making in the implementation of optimization strategies in urban agricultural systems. In terms of experimental design, further research assessing the nutritional flows of cascade systems should increase the number of plants that will be analyzed in terms of nutrients to precisely determine the variability of concentrations within the same treatment and test.

## Data Availability Statement

The raw data supporting the conclusions of this article will be made available by the authors, without undue reservation.

## Author Contributions

MR-S, AP-B, GV, and XG conceived the original idea for the study. MR-S, VA-P, and FP set up, supervised, and acquired the data for the experimental tests. MR-S processed and analyzed the data and took the lead in writing the manuscript. All authors were responsible for the conception and design of the study, critically revised the draft for important intellectual content, and gave their final approval to the manuscript.

## Conflict of Interest

The authors declare that the research was conducted in the absence of any commercial or financial relationships that could be construed as a potential conflict of interest.
