# Supplementary material for: Closed-Loop Crop Cascade to Optimize Nutrient Flows and Grow Low-Impact Vegetables in Cities
Source: Front Plant Sci. 2020 Nov 12;11:596550. doi: 10.3389/fpls.2020.596550 (PMC7688993; doi:10.3389/fpls.2020.596550)
Supplement: Supplementary file 1 [file Data_Sheet_1.DOCX]

**SUPPLEMENTARY MATERIAL**

**Closed-loop crop cascade to optimize nutrient flows and grow low-impact vegetables in cities**

Martí Rufí-Salís^1,2,*^, Felipe Parada^1^, Verónica Arcas-Pilz^1^, Anna Petit-Boix^3^, Gara Villalba^1,2^, Xavier Gabarrell^1,2^

^1^Sostenipra Research Group (2017 SGR 1683), Institut de Ciència i Tecnologia Ambientals (ICTA-UAB), María de Maeztu Unit, Universitat Autònoma de Barcelona (UAB), 08193 Cerdanyola del Vallès, Barcelona, Spain

^2^Department of Chemical, Biological and Environmental Engineering, Universitat Autònoma de Barcelona (UAB), Campus UAB, 08193 Bellaterra, Barcelona, Spain

^3^Chair of Societal Transition and Circular Economy, University of Freiburg. Tennenbacher Str. 4, 79106 Freiburg i. Br. (Germany)

*Corresponding author: Marti.Rufi@uab.cat


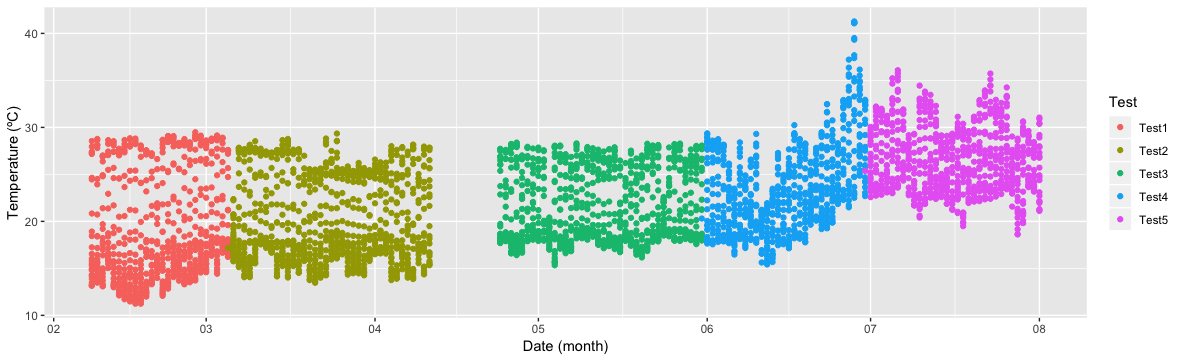


Figure S1. Hourly temperatures for the 5 tests


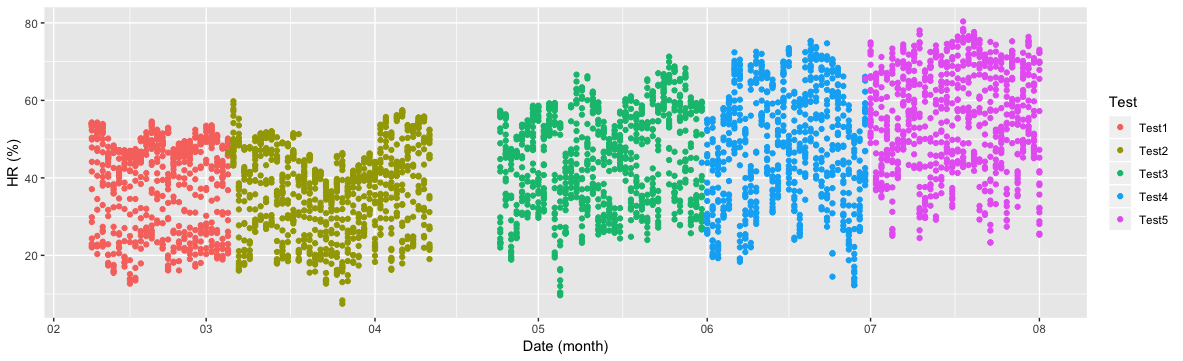


Figure S2. Hourly relative humidity values for the 5 tests

Figure S3. pH


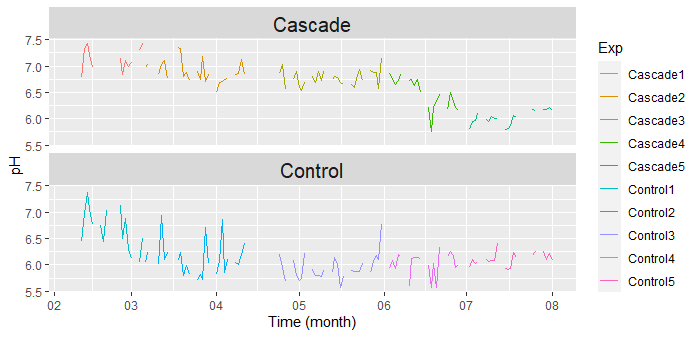


| Table S1. Uptaken nutrients per plant in the receiving crop (mg) | | | | | | | | |
| --- | --- | --- | --- | --- | --- | --- | --- | --- |
| Nutrients | T1 - Cas | T2 - Cas | T3 - Cas | T3 - Con | T4 - Cas | T4 - Con | T5 - Cas | T5 - Con |
| N | 89.8 | 118.1 | 155.7 | 362.9 | 397.7 | 419.5 | 235.3 | 324.6 |
| P | 26.3 | 28.3 | 29.1 | 86.6 | 85.8 | 85.4 | 42.1 | 58.1 |
| K | 352.9 | 396.6 | 459.4 | 1233.7 | 1007.6 | 1026.4 | 541.3 | 755.2 |
| Ca | 70.8 | 53.1 | 53.6 | 134.6 | 145.4 | 143.8 | 69.4 | 110.3 |
| Mg | 13.2 | 11.0 | 12.2 | 27.1 | 30.6 | 27.3 | 18.1 | 23.6 |
| S | 10.4 | 12.4 | 13.1 | 34.8 | 30.5 | 32.5 | 17.4 | 23.5 |

| Table S2. Irrigated nutrients per plant in the receiving crop (mg) | | | | | | | | |
| --- | --- | --- | --- | --- | --- | --- | --- | --- |
| Nutrients | T1 - Cas | T2 - Cas | T3 - Cas | T3 - Con | T4 - Cas | T4 - Con | T5 - Cas | T5 - Con |
| N | 62.4 | 101.9 | 361.4 | 2117.9 | 1708.0 | 2558.8 | 3599.4 | 1941.6 |
| P | 94.4 | 312.8 | 333.2 | 855.6 | 1341.0 | 1236.9 | 2245.9 | 1170.4 |
| K | 556.9 | 1628.5 | 2640.0 | 3874.4 | 7538.7 | 5190.2 | 10704.5 | 4731.8 |
| Ca | 299.1 | 771.5 | 997.0 | 2945.7 | 2876.8 | 3395.2 | 5022.7 | 2727.6 |
| Mg | 86.6 | 170.4 | 193.7 | 332.7 | 486.5 | 372.1 | 740.2 | 292.2 |
| S | 229.0 | 757.2 | 1157.2 | 1085.7 | 2338.4 | 1087.6 | 2889.4 | 1196.1 |

| Table S3. Leached nutrients per plant in the receiving crop (mg) – calculated through the difference between Table S2 and S1. | | | | | | | | |
| --- | --- | --- | --- | --- | --- | --- | --- | --- |
| Nutrients | T1 - Cas | T2 - Cas | T3 - Cas | T3 - Con | T4 - Cas | T4 - Con | T5 - Cas | T5 - Con |
| N | 0.0 | 0.0 | 205.8 | 1755.0 | 1310.3 | 2139.3 | 3364.2 | 1617.0 |
| P | 68.1 | 284.5 | 304.1 | 769.0 | 1255.2 | 1151.5 | 2203.8 | 1112.2 |
| K | 204.0 | 1231.9 | 2180.7 | 2640.7 | 6531.2 | 4163.8 | 10163.1 | 3976.6 |
| Ca | 228.3 | 718.4 | 943.4 | 2811.1 | 2731.4 | 3251.3 | 4953.3 | 2617.3 |
| Mg | 73.4 | 159.5 | 181.5 | 305.6 | 455.9 | 344.8 | 722.1 | 268.6 |
| S | 218.6 | 744.8 | 1144.0 | 1050.9 | 2307.9 | 1055.2 | 2872.0 | 1172.5 |
